# Clinical value of comprehensive genomic profiling on clinical trial enrollment for patients with advanced solid tumors

**DOI:** 10.1093/oncolo/oyae293

**Published:** 2024-10-29

**Authors:** Richard S P Huang, Jessica K Lee, Katherine T Lofgren

**Affiliations:** Foundation Medicine, Inc., Cambridge, MA, United States; Foundation Medicine, Inc., Cambridge, MA, United States; Foundation Medicine, Inc., Cambridge, MA, United States

**Keywords:** clinical value, CGP, clinical trial enrollment, advanced solid tumor

## Abstract

The use of biomarker testing to inform treatment decisions has emerged as a standard of care in multiple cancer types. However, the rates of patients with genomic testing results in hand to inform treatment decision-making remain variable. Here, we studied the impact of comprehensive genomic profiling (CGP) on clinical trial enrollment rates in patients with advanced-stage non-small cell lung, colorectal, breast, and prostate cancer using a real-world clinicogenomic database. On average, clinical trial enrollment in the therapy line immediately after CGP report receipt was 5.4%, which represents a 3.0 percentage point increase compared to therapy lines preceding CGP report receipt, supporting a meaningful association between CGP report availability and increased clinical trial enrollment.

## Introduction

In the past decade, a paradigm shift in the treatment of oncology patients has occurred. There are now a plethora of biomarker-specific therapeutic agents approved by the United States Food and Drug Administration (FDA) with an accompanying companion diagnostic (CDx).^1^ The use of comprehensive genomic profiling (CGP) assays that can detect multiple genomic biomarkers simultaneously and efficiently has emerged as the optimal precision oncology standard-of-care to select among FDA-approved therapies and for clinical trial enrollment.^2,3^ In addition, liquid biopsy-based CGP has increased in popularity as an assay modality due to the relative ease of obtaining a liquid biopsy.^4^ While the ability to detect targetable genomic alterations with CGP has been well characterized, the association of CGP testing with clinical trial enrollment has not been well characterized to date. Here, we quantify the clinical value of CGP testing on clinical trial enrollment through a retrospective analysis of advanced-stage non-small cell lung (NSCLC), colorectal (CRC), breast, or prostate cancer utilizing a nationwide (US-based) de-identified clinico-genomic database.^5^

## Methods

A retrospective analysis of advanced-stage non-small cell lung, colorectal, breast, and prostate cancer was performed using the nationwide (US-based) de-identified Flatiron Health (FH)-Foundation Medicine (FMI) clinico-genomic database (CGDB). The de-identified data originated from approximately 280 US cancer clinics (~800 sites of care). Retrospective longitudinal clinical data were derived from electronic health record data, comprising patient-level structured and unstructured data, curated via technology-enabled abstraction, and were linked to genomic data derived from FMI comprehensive genomic profiling tests by de-identified, deterministic matching. The study cohort was limited to patients with either a FoundationOneCDx or FoundationOneLiquid CDx report delivered between January 2018 and March 2024. Specifically, we examined clinical trial participation pre- and post-CGP report delivery over time. We also studied post-report enrollment trends by biopsy type, treatment line number, and site of care (academic vs. community care).

## Results

**Figure 1.**
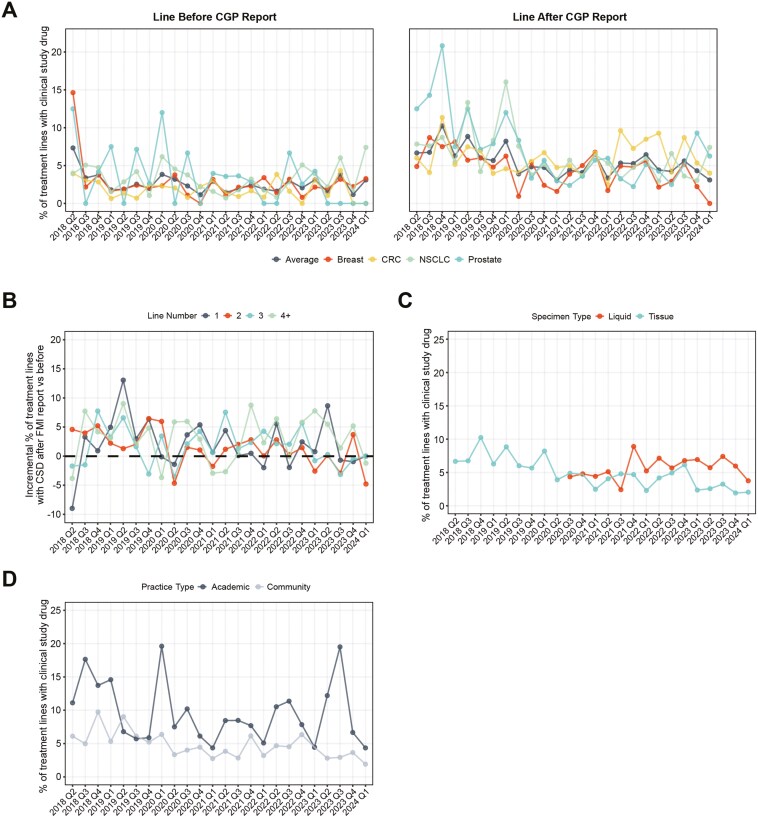
Clinical trial enrollment between 2018 and 2024. Percent of treatment lines containing a clinical study drug relative to (A) CGP report availability, (B) line of therapy number, (C) biopsy type, and (D) site of care. % of treatment lines containing a clinical study drug by biopsy site, site of care, and line number reflect lines given after CGP report receipt.

We analyzed 9689 advanced or metastatic NSCLC, CRC, breast, and prostate cancer patients with documented lines of therapy received before and after CGP report receipt (Figure S1). On average, clinical trial enrollment in the therapy line immediately after CGP report delivery was 5.4%. We discovered that compared to the therapy line prior to CGP-report delivery, post-report enrollment was incrementally higher by 124% (a 3.0 percentage point increase, *P* < .001) which remained consistent over time (Figure 1A, Figure S2). While post-report enrollment rates were higher in patients treated in later lines of therapy (4.2%, 4.8%, 6.3%, and 6.9% in first through fourth lines respectively, *P* = .0008), the incremental increase in clinical study drug use after CGP report delivery remained consistent across therapy line numbers (Figure 1B, Figure S3). Post-report enrollment rates were consistent regardless of tissue vs blood-based CGP (5.3% vs 5.9%, *P* = .31) (Figure 1C, Figure S4), but enrollment rates among patients were higher in academic centers compared to community practices (9.3% and 4.0%, *P* < .0001) (Figure 1D).

## Discussion

This work supports a meaningful association between CGP report availability and increased clinical trial enrollment across major oncologic tumor types. While CGP seems to enable increased clinical trial enrollment, a limitation of this work is our inability to differentiate between clinical trial enrollment after CGP in the course of routine clinical care vs testing as a trial enrollment prerequisite. Additionally, cohort sizes for certain diseases and population subgroups such as prostate and patients treated in academic settings were small and our findings would need to be confirmed in larger cohorts. In addition, the results here affirm the usage of liquid biopsies to enable clinical study enrollment. Unsurprisingly, academic centers had higher rates of clinical trial enrollment, suggesting that there is more to be done to make clinical trials available to all segments of the population.^6^

## Supplementary material

Supplementary material is available at *The Oncologist* online.

oyae293_suppl_Supplementary_Figures_1-4

## Data Availability

The authors declare that all relevant aggregate data supporting the findings of this study are available within the article and its supplementary information files. The data that support the findings of this study originated from Flatiron Health, Inc. and Foundation Medicine, Inc. Requests for data sharing by license or by permission for the specific purpose of replicating results in this manuscript can be submitted to PublicationsDataaccess@flatiron.com and cgdb-fmi@flatiron.com. In accordance with the Health Insurance Portability and Accountability Act, we do not have IRB approval or patient consent to share individualized patient genomic data, which contains potentially identifying or sensitive patient information and cannot be reported in a public data repository.
