# Supplementary material for: Clinical value of comprehensive genomic profiling on clinical trial enrollment for patients with advanced solid tumors
Source: Oncologist. 2024 Oct 29;30(7):oyae293. doi: 10.1093/oncolo/oyae293 (PMC12311268; doi:10.1093/oncolo/oyae293)

**Supplemental Data**

**Supplemental Figure 1: Consort Diagram**


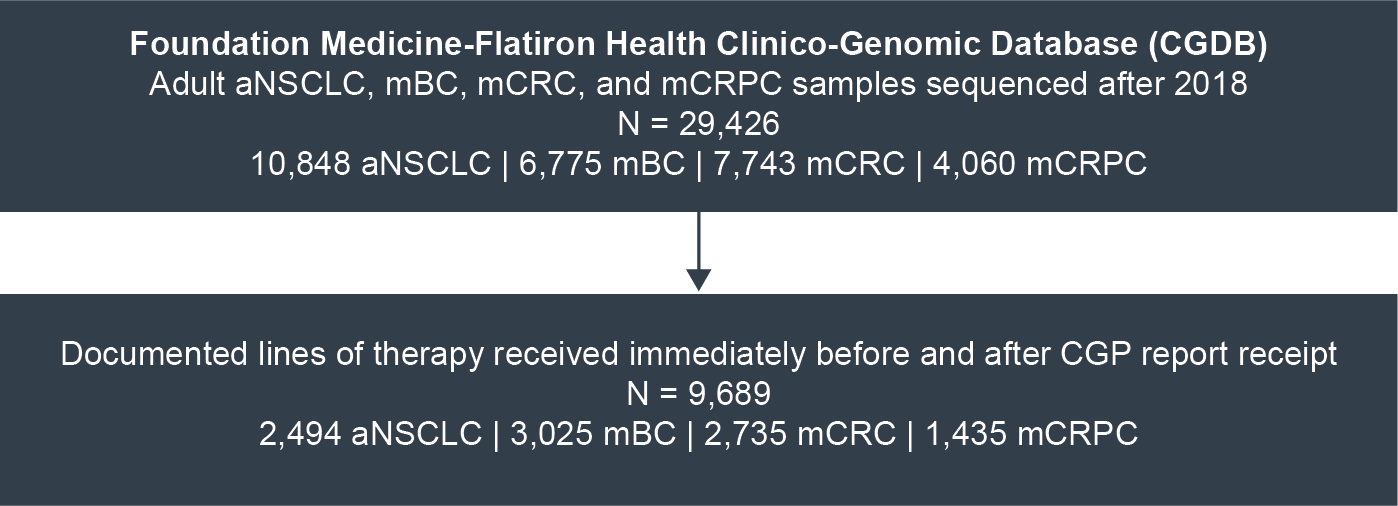


aNSCLC=advanced non-small cell lung cancer, mBC=metastatic breast cancer, mCRC=metastatic colorectal carcinoma, mCRPC=metastatic castrate resistant prostate cancer

**Supplemental Figure 2:** Incremental percent of treatment lines after vs. before CGP report containing a clinical trial drug


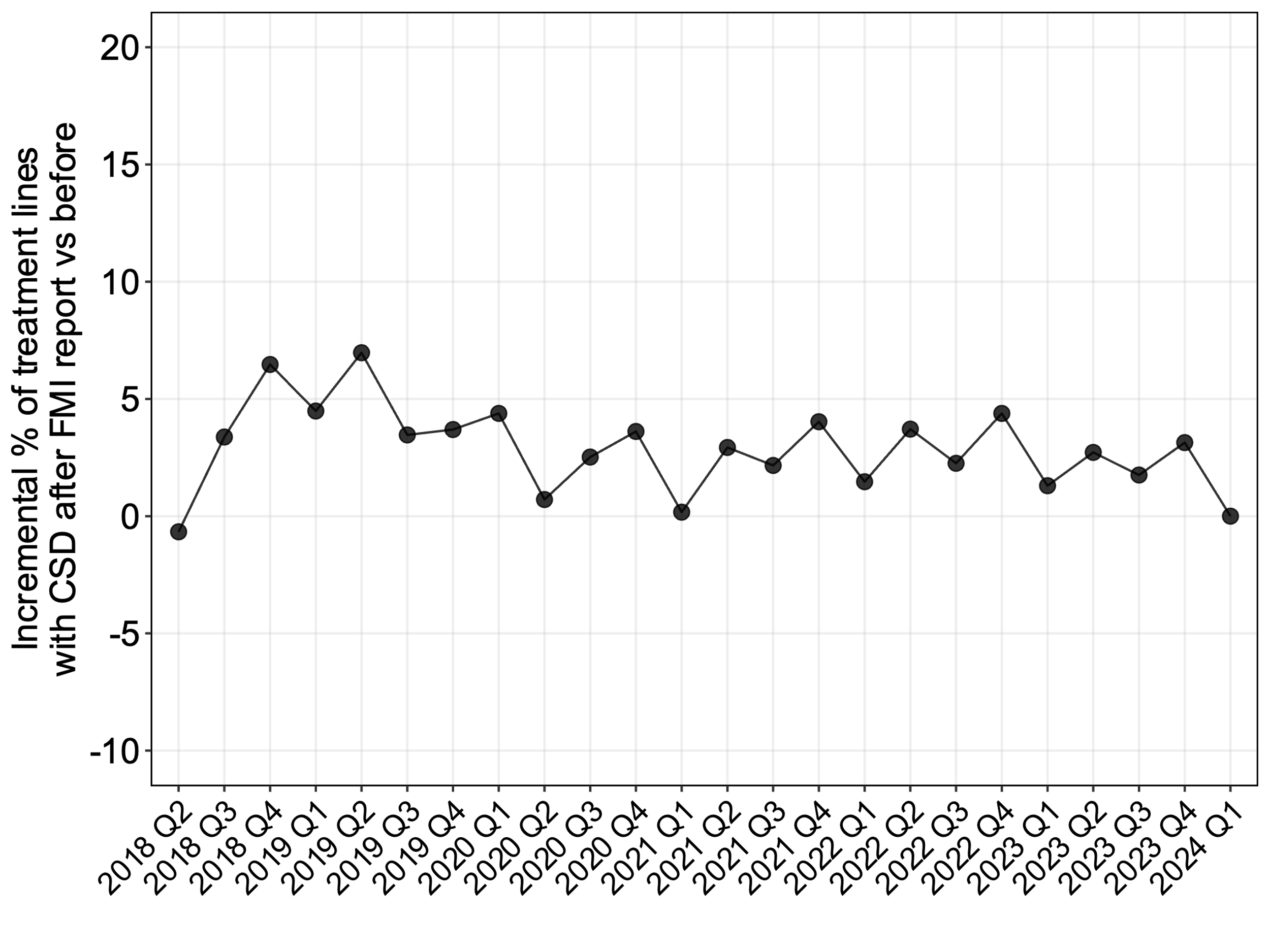


**Supplemental Figure 3:** Incremental percent of treatment lines after vs. before CGP report containing a clinical study drug by line number and tumor type


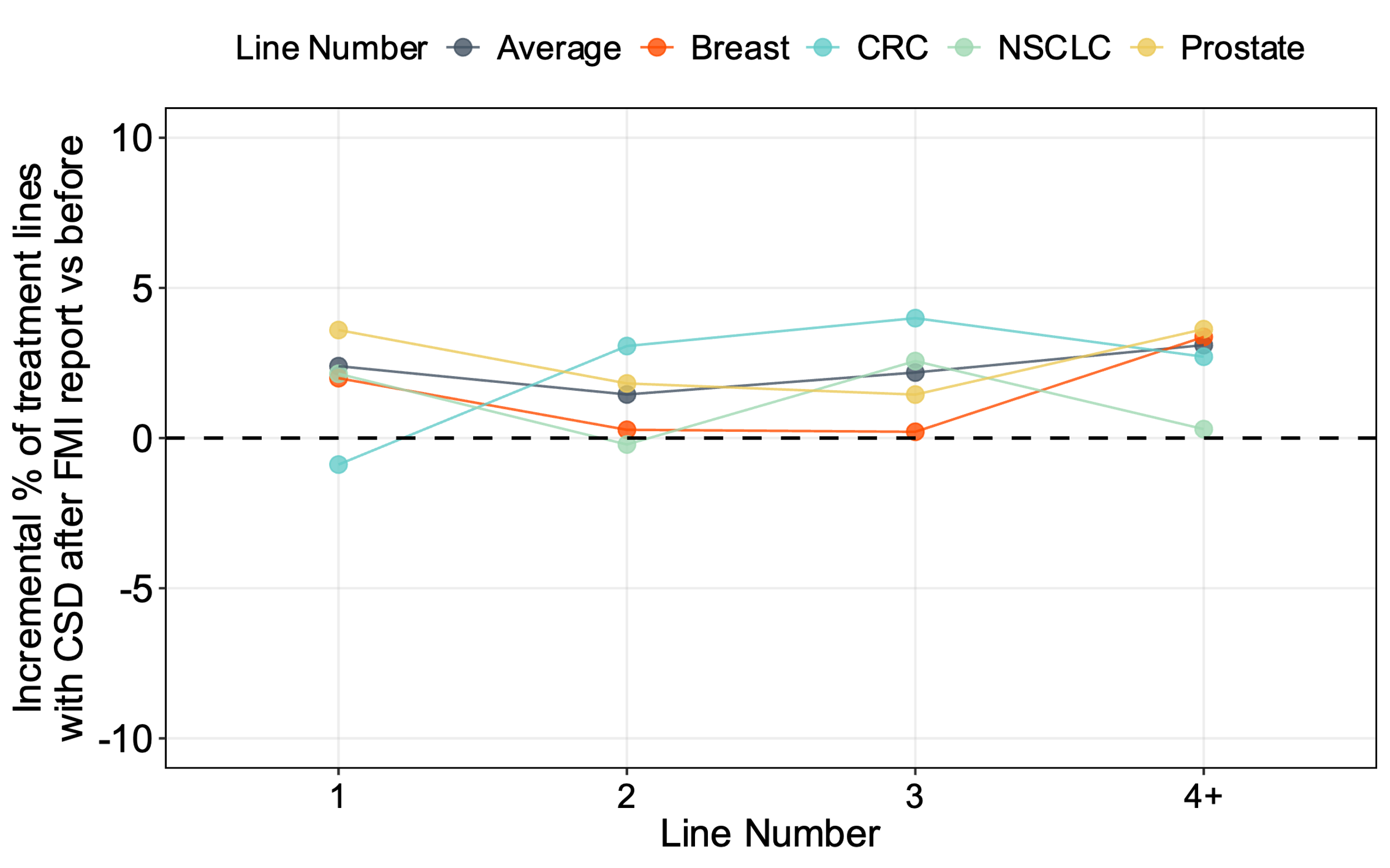


**Supplemental Figure 4:** Percent of treatment lines containing a clinical study drug after CGP report receipt by tumor type


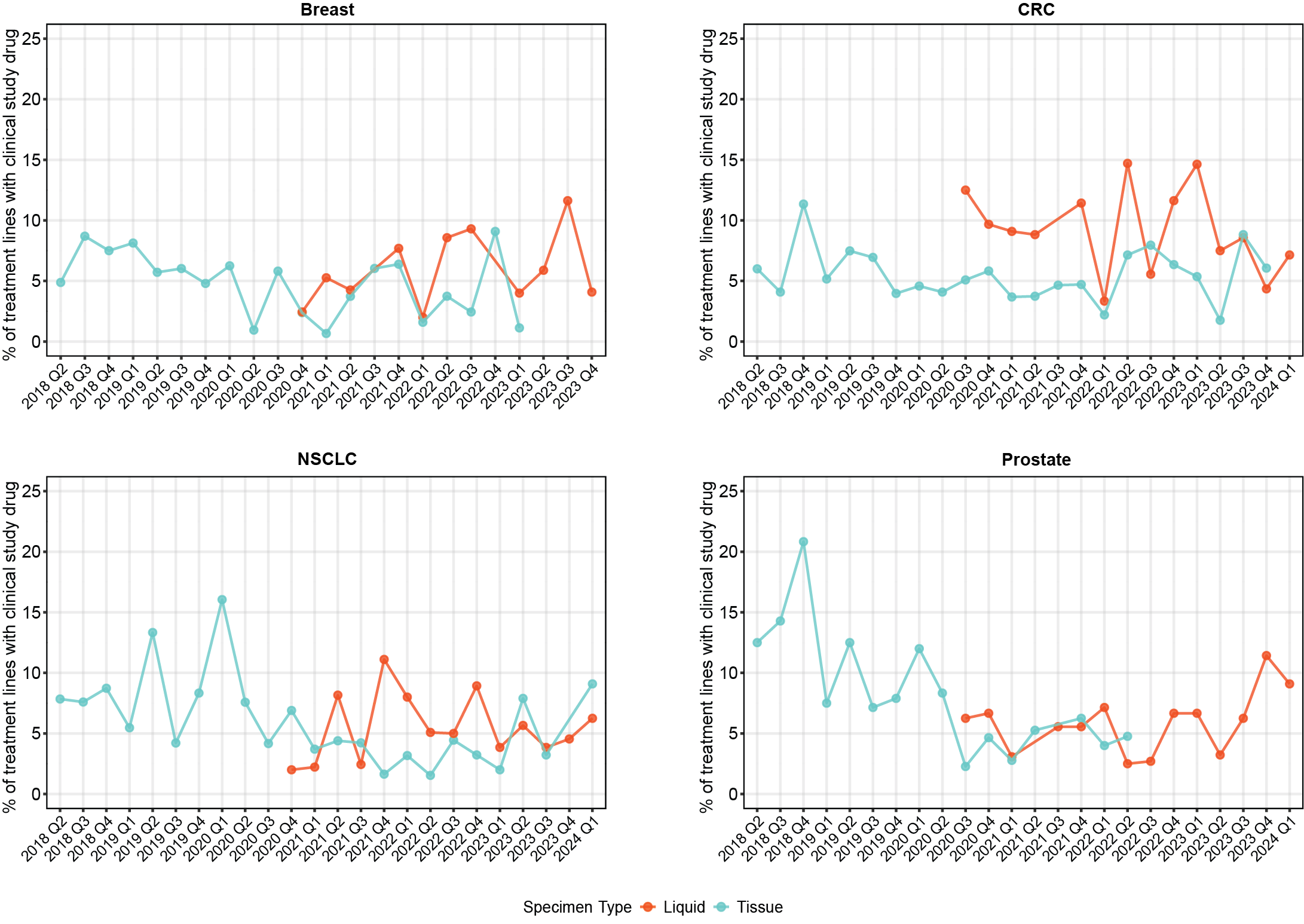

Supplement: oyae293_suppl_Supplementary_Figures_1-4 [file oyae293_suppl_supplementary_figures_1-4.docx]
